# Stable Deep Neural Network Architectures for Mitochondria Segmentation on Electron Microscopy Volumes

**DOI:** 10.1007/s12021-021-09556-1

**Published:** 2021-12-02

**Authors:** Daniel Franco-Barranco, Arrate Muñoz-Barrutia, Ignacio Arganda-Carreras

**Affiliations:** 1 Donostia International Physics Center (DIPC), Donostia-San Sebastián, Spain; 2 Department of Computer Science and Artificial Intelligence, University of the Basque Country (UPV/EHU), Donostia-San Sebastian, Spain; 3 Universidad Carlos III de Madrid, Leganés, Spain; 4 Instituto de Investigación Sanitaria Gregorio Marañón, Madrid, Spain; 5 Ikerbasque, Basque Foundation for Science, Bilbao, Spain

**Keywords:** Electron microscopy, Mitochondria, Semantic segmentation, Deep learning, Bioimage analysis

## Abstract

Electron microscopy (EM) allows the identification of intracellular organelles such as mitochondria, providing insights for clinical and scientific studies. In recent years, a number of novel deep learning architectures have been published reporting superior performance, or even human-level accuracy, compared to previous approaches on public mitochondria segmentation datasets. Unfortunately, many of these publications make neither the code nor the full training details public, leading to reproducibility issues and dubious model comparisons. Thus, following a recent code of best practices in the field, we present an extensive study of the state-of-the-art architectures and compare them to different variations of U-Net-like models for this task. To unveil the impact of architectural novelties, a common set of pre- and post-processing operations has been implemented and tested with each approach. Moreover, an exhaustive sweep of hyperparameters has been performed, running each configuration multiple times to measure their stability. Using this methodology, we found very stable architectures and training configurations that consistently obtain state-of-the-art results in the well-known EPFL Hippocampus mitochondria segmentation dataset and outperform all previous works on two other available datasets: Lucchi++ and Kasthuri++. The code and its documentation are publicly available at https://github.com/danifranco/EM_Image_Segmentation.

**Supplementary Information:**

The online version contains supplementary material available at 10.1007/s12021-021-09556-1.

## Introduction

Recent imaging methods in electron microscopy (EM) allow scientists to identify subcellular organelles such as vesicles or mitochondria with nano-scale precision. Mitochondria play an important role in some crucial functions in the cell, such as energy production, signaling, differentiation, cell growth and death (Tait & Green, 2012). For that reason, the automated and accurate segmentation of mitochondria is especially relevant for basic research in neuroscience, but in clinical studies as well, since their number and morphology are related to severe diseases such as cancer (De Moura et al., 2010; Fulda et al., 2010; Wallace, 2012), Parkinson (Poole et al., 2008) or Alzheimer disease (De Moura et al., 2010).

In the past decade, advances in computer vision, especially those based on deep learning (DL), have helped scientists to automatically quantify the size and morphology of cells and organelles in microscopy images (Moen et al., 2019; Meijering, 2020). However, with an increasing number of DL-based bioimage segmentation publications every year, there is a lack of enough benchmarks for different image modalities and segmentation problems to compare state-of-the-art methods under the same conditions. Moreover, DL methods are usually too data-specialized, making it difficult to identify those approaches that perform well on datasets different from those they have been tested on (Isensee et al., 2021). On top of that, many of such approaches are published without their supporting code and image data, leading to major reproducibility and reliability problems. Such issues have not gone unnoticed. They have become the main target even for recently proposed challenges (https://paperswithcode.com/rc2020) where the machine learning community aims at reproducing the computational experiments and verifying the empirical results already published at top venues.

As pointed out by recent works (Bello et al., 2021; Isensee et al., 2021), while many publications insist on presenting architectural novelties, the overall performance of a network depends substantially on its corresponding pre-processing, training, inference and post-processing strategies. Even though such choices play a critical role in the final results, very often they tend to be omitted in the method descriptions and their comparisons with competing approaches. Another issue inherent to the use of deep learning architectures (and frequently not discussed in publications) is the sometimes not negligible variability of the results produced by different executions of the same architecture and training configuration. Despite programmatically setting all initial random seeds, the non-deterministic nature of the graphical processing units (GPUs) introduces variations from execution to execution, resulting in slightly different performances. This variability is usually not taken into account when presenting results, although it could be crucial to select models, training, and inference strategies that repeatedly lead to stable results.

In the particular task of mitochondria segmentation, the *de facto* benchmark dataset adopted by the community is the EPFL Hippocampus dataset (Lucchi et al., 2011) (hereafter referred to as Lucchi dataset). Published in 2011, it contains two image volumes (training and test) of the same size, and their respective semantic segmentation labels are both public. As the reference in the field for a decade, many methods have been published proposing solutions for this dataset. Unfortunately, most of them suffer from the aforementioned problems, forcing other scientists to code their own versions of the published algorithms, often knowing too few details about their original implementations, training, and inference methodologies.

To address these deficiencies in the field, we first re-implemented the top-performing DL architectures for the Lucchi dataset following the descriptions of their original publications. After our own modifications, an extensive hyperparameter search, and multiple runs of the same configuration, some of these methods occasionally achieved their claimed results. Next, we compared the performance of state-of-the-art biomedical semantic segmentation architectures in the same dataset, evaluated under the same training and inference framework. In particular, we focused on the stability of the resulting metric values after several executions of the same configuration and scrutinized the impact of different popular post-processing and output reconstruction methods. Finally, based on our findings, we propose light encoder-decoder architectures that consistently lead to robust state-of-the-art results in Lucchi as well as in other public mitochondria segmentation datasets.

In brief, our main contributions are as follows:

1. We performed a thorough study on the reproducibility and stability of the top-performing DL segmentation methods published for the Lucchi dataset, exposing major issues to consistently achieve their claimed results.
2. We made a comprehensive comparison of the performance of the most popular deep learning architectures for biomedical segmentation using the Lucchi dataset, and show their stability under the same training and post-processing conditions.
3. We propose different variations of light-weight encoder-decoder architectures, together with a training/inference workflow, that lead to stable and robust results across mitochondria segmentation datasets.

## Related Work

In the last decade, DL approaches have become dominant in the most common target applications of computer vision (Garcia-Garcia et al., 2018; Minaee et al., 2021) including semantic segmentation for biomedical images (Haque & Neubert, 2020; Litjens et al., 2017). Semantic segmentation aims at associating each pixel in an image to a class label. The first steps towards resolving this problem using DL were taken by means of fully convolution networks (FCNs) (Long et al., 2015). More specifically, fully connected layers were replaced by convolutional layers in some classic networks (Krizhevsky et al., 2012; Simonyan & Zisserman, 2014; Szegedy et al., 2015) and information from intermediate layers was fused to upsample the feature maps encoded by the network, producing a pixel-wise classification. This idea of *encoding* the image through a convolutional neural network (CNN), outputting a vector feature map (also called *bottleneck*), and recovering its original spatial shape in a *decoding* path was further extended in subsequent works (Noh et al., 2015; Ronneberger et al., 2015; Milletari et al., 2016; Jégou et al., 2017; Badrinarayanan et al., 2017; Chaurasia & Culurciello, 2017). A major breakthrough was the U-Net (Ronneberger et al., 2015), which extended the encoding and decoding idea by making an upsampling path with up-convolutions after the bottleneck to recover the original image size. In addition, the authors proposed skip connections between the contracting and the expanding path, allowing the upsampling path to recover fine-grained details. The U-Net is the baseline of numerous approaches due to its success in multiple biomedical applications (Zhou et al., 2018; Schlemper et al., 2019; Roy et al., 2018; Arganda-Carreras et al., 2015; Gu et al., 2019; Buhmann et al., 2018; Ibtehaz & Rahman, 2020; Zhuang, 2018; Jin et al., 2019).

In the specific case of mitochondria segmentation, early works attempting to segment the Lucchi dataset (Lucchi et al., 2011) leveraged traditional image processing and machine learning techniques (Lucchi et al., 2012, 2013, 2014a, b). In their last two works, Lucchi et al. (2014a, b) proposed alternative methodologies to segment mitochondria on their own dataset explicitly modeling their membranes. From those results, Casser et al. (2020) inferred a Jaccard index or intersection over union (IoU) lower bound value of 0.895 in the test set. The IoU is a common way of measuring the overlapping area between the ground truth and the produced segmentation with values that range from 0 to 1, where 1 represents a perfect match (see “Experimental setup”).

More modern approaches made use of DL architectures to segment the Lucchi dataset. For instance, Oztel et al. (2017) trained a CNN with four convolutional layers to classify $32\times 32$ pixel patches extracted from the training data into mitochondria and background. After that, they fed the network with the full test images to simulate a sliding window process and applied three consecutive post-processing methods: 1) spurious detection to remove small false blobs, 2) marker-controlled watershed transform (Meyer, 1994) for border refinement, and 3) median filtering to smooth labels along the z-axis. This way, they reported an IoU value of 0.907 in the test set, which is the highest value to date. Liu et al. (2018) used instead a modified Mask R-CNN (He et al., 2017) to detect and segment mitochondria. As post-processing methods they performed: 1) a morphological opening to eliminate small regions and smooth large ones, 2) a multi-layer fusion operation to exploit 3D mitochondria information, and 3) a size-based filtering to remove tiny segments that have an IoU score below a given threshold. As a result, they reported an IoU value of 0.849 in the test set. Cheng and Varshney (2017) applied both a 2D and a 3D version of an asymmetric U-Net-like network. They introduced the *stochastic downsampling* method, an operation they named *feature level augmentation*. More specifically, on that downsampling layer, they subdivided the image into fixed square regions and picked random rows and columns inside them to select the pixels/voxels that will constitute the downsampled output. Moreover, they implemented factorized convolutions (Szegedy et al., 2016) instead of classical ones to drastically reduce the number of network parameters. As their best result, they reported an IoU value of 0.889 in the test set using their 3D network. Xiao et al. (2018) employed a variant of a 3D U-Net model with residual blocks. In the decoder of the network, they included two auxiliary outputs to address the vanishing gradient issue. Their final output is the result of the ensemble prediction of the 16 possible 3D variations (using flips and axis rotations) per each 3D subvolume. They reported an IoU value of 0.900 in the test set. In a more recent work, Casser et al. (2020) presented a light version of a 2D U-Net aiming to achieve real-time segmentation and reported an IoU value of 0.890 applying median *Z-filtering* as post-processing method.

**Fig. 1:**
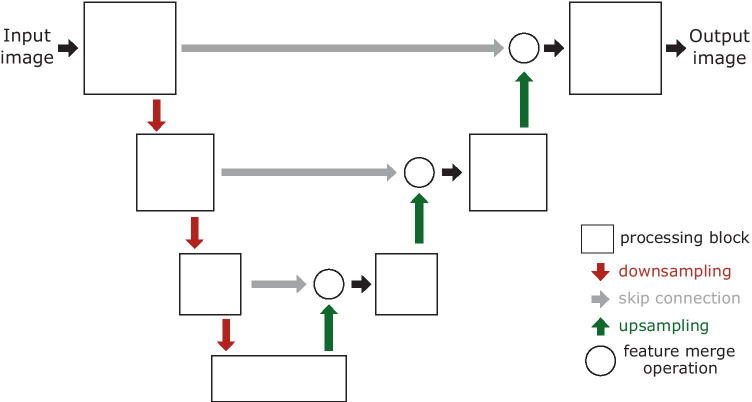
Graphical representation of the proposed network architectures. Depending on the model of choice, the processing blocks can be either simply convolutional or residual blocks, while the feature merge operations may imply a single concatenation or an additional attention gate

**Fig. 2:**
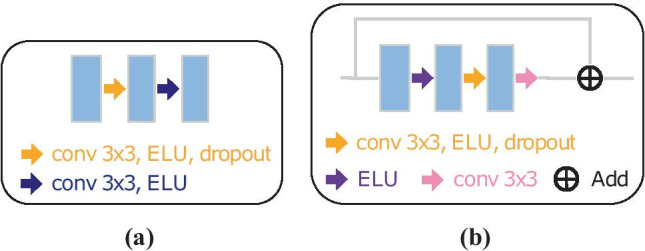
Types of processing blocks. Convolutional blocks **(a)** are used in the U-Net and Attention U-Net architectures, and residual blocks **(b)** are used in the Residual U-Net

## Methods

Although architectural modifications of a basic U-Net to perform biomedical segmentation are continuously published, it is usually unclear if their claimed superiority is only due to an incomplete optimization of the basic network for the task at hand (Isensee et al., 2021; Bello et al., 2021). We hypothesize that, on top of answering that question, a full optimization can also lead to lightweight models that constantly produce stable and robust results across datasets. To prove it, we explored basic U-Net configurations together with popular architectural tweaks such as residual connections (He et al., 2016a) or attention gates (Schlemper et al., 2019). Additionally, to disentangle the impact of each training choice, all configurations are run several times and their results are shown in the context of different post-processing and output reconstruction methods.

### Proposed Networks

**Fig. 3:**
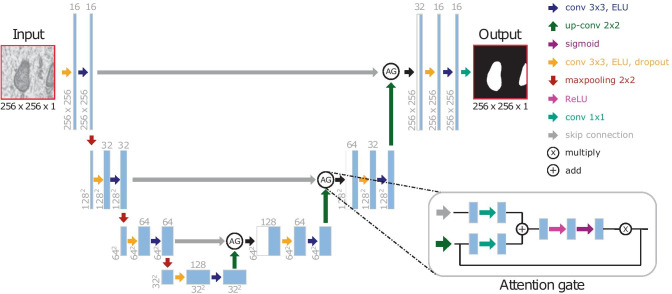
Proposed 2D Attention U-Net architecture. Example with three downsampling levels and a detailed description of the attention gates used in the skip connections

Building upon the state of the art, we have explored different lightweight U-Net-like architectures in 2D and 3D. The general scheme is represented in Fig. 1, where our basic and Attention U-Net models use convolutional blocks as processing blocks (two $3\times 3$ convolutional layers, Fig. 2a) and our Residual U-Net is formed by full pre-activation (He et al., 2016b) residual blocks (two $3\times 3$ convolutional layers with a shortcut, Fig. 2b). Both basic and Residual U-Net use concatenation as feature merge operation while our Attention U-Net introduces there an attention gate (Schlemper et al., 2019). Based on a thorough hyperparameter exploration (see supplementary material), we found the following optimal configuration for each architecture:

- **Basic U-Net**. In 2D, it is a four-level U-Net with 16 filters in the initial level that get doubled on each level, dropout in each block (from 0.1 up to 0.3 in the bottleneck and reversely, from 0.3 to 0.1 in the upsampling layers), ELU activation functions and transposed convolutions to perform the upsampling in the decoder. In 3D, the architecture is very similar, but using 3 levels, with 28, 36, 48 and 64 (in the bottleneck) 3D filters on each layer.
- **Residual U-Net**. In 2D, this network is identical to our best basic U-Net architecture but swapping each convolutional block by a residual block (He et al., 2016a). For the 3D residual approach, we achieved our best results going one level deeper than the non-residual 3D network and 28, 36, 48, 64 and 80 (bottleneck) filters per level.
- **Attention U-Net**. These networks are the same as Basic U-Net but incorporating attention gates (Schlemper et al., 2019) in the features passed by the skip connections (Fig. 3). Such attention mechanism emphasizes salient feature maps that are in charge of the class decision and suppress irrelevant ones endowing the network with the ability to focus on relevant regions of the image.

### Post-Processing

**Fig. 4:**
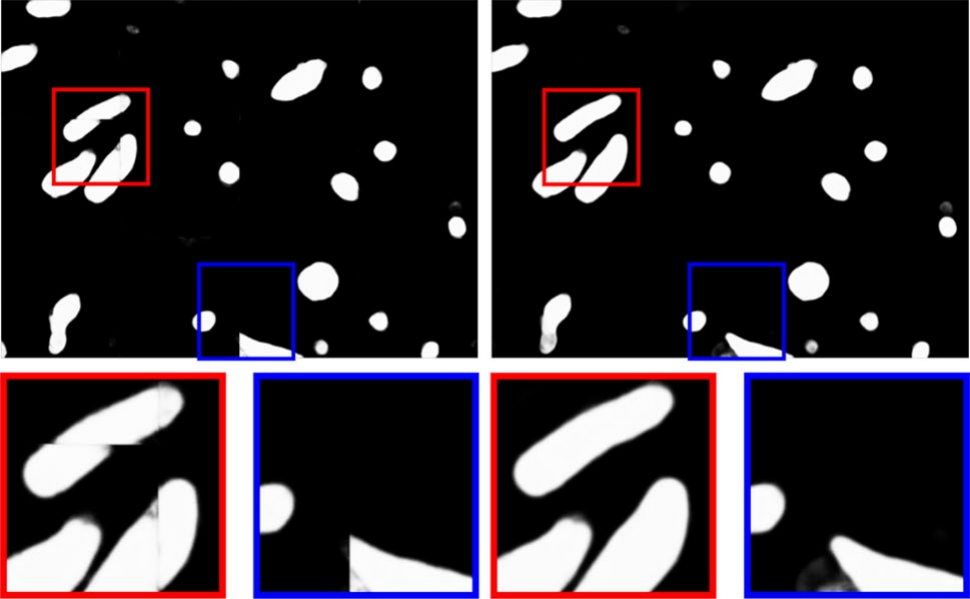
Border effect in output image reconstruction. From left to right: output image reconstructed from patches with visible jagged predictions; and output image reconstructed using both the blending and ensemble techniques. Blue and red boxes show zoomed areas on both images

As the network outputs are pixel-wise predictions, it is common practice to apply basic post-processing methods to improve the results. We experimented with three techniques and studied their impact in the final segmentation result:

- **Test-time data augmentation.** Inference is applied on the multiples of $90^\circ$ rotations and flipped versions of each image. Consequently, eight versions are created in 2D and 16 versions in 3D. Finally, the individual transformations are undone and the results are averaged into a final prediction for an ensemble effect.
- **Blending overlapped patches.** When networks work on image patches, the final prediction is reconstructed as a mosaic of the patches predictions. The presence of jagged predictions on the borders of the output patches are a recurrent problem (Fig. 4) that can be mitigated by creating overlapping patches and smoothly blending the resulting predictions using a second order spline window function. Due to its computational cost, we only experimented with this technique in 2D.
- **Median Z-filtering.** A simple median filter along the Z-axis (Casser et al., 2020; Oztel et al., 2017) can be used to correct label predictions in consecutive image slices.

### Output Reconstruction

During the training of deep networks, the input images are commonly divided into patches due to GPU memory limitations. Later, those patches need to be merged back together to form the final output at full-image size. In some publications, the authors specify clearly the way they infer and merge their predictions (Xiao et al., 2018), while in others this process is not described (Cheng & Varshney, 2017; Oztel et al., 2017; Casser et al., 2020), hindering a direct comparison between methods’ performance. Following the code of good practices to show deep learning-based results proposed by Dodge et al. (2019), all results presented in this paper state the reconstruction strategy used. Namely, the implemented options are as follows:

1. **Per patch**. The metric value corresponds to the average value over all patches.
2. **Per image (with** $50\%$ **overlap)**. The patches are merged together using $50\%$ of overlap and the metric value is the average overall reconstructed images.
3. **Full image**. Inference is applied on the full-sized images. The metric value is the average over all images. This strategy is not always feasible, since it depends on the input image size and the available GPU memory.

## Experimental Results

To test our hypothesis and focusing on model reproducibility and stability, we conducted a thorough study on the top-performing segmentation methods recently published in the Lucchi dataset. Additionally, we introduce our own solutions, compare them with state-of-the-art approaches in biomedical semantic segmentation and test them in other public datasets. In all our experiments, we present average scores obtained running the same configuration 10 times (hereafter referred as a *run*) together with the corresponding standard deviation.

### Datasets

**Fig. 5:**
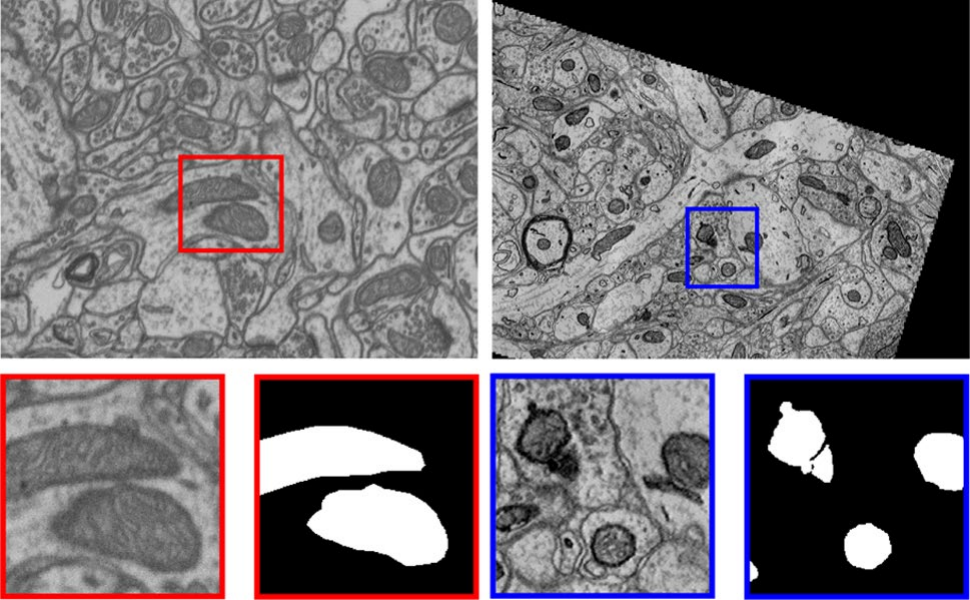
Sample images from public mitochondria datasets. From left to right: Lucchi and Kasthuri++ data sample with their corresponding binary mask. Blue and red boxes show zoomed areas on both images

All the experiments performed in this work are based on the following publicly available datasets:

**EPFL Hippocampus or Lucchi dataset** (Lucchi et al., 2011). The original volume represents a $5\times 5\times 5$ $\mu m$ section of the CA1 hippocampus region of a mouse brain, with an isotropic resolution of $5\times 5\times 5$ nm per voxel. The volume of $2048\times 1536\times 1065\,\text{ voxels }$ was acquired using focused ion beam scanning electron microscopy (FIB-SEM). The mitochondria of two subvolumes formed by 165 images of $1024\times 768$ pixels were manually labeled by experts (Fig. 5 (red)), and are commonly used as training and test data.

**Lucchi++ dataset** (Casser et al., 2020). This is a version of the Lucchi dataset after two neuroscientists and a senior biologist re-labeled mitochondria by fixing misclassifications and boundary inconsistencies.

**Kasthuri++ dataset** (Casser et al., 2020). This is a re-labeling of the dataset by Kasthuri et al. (2015) (Fig. 5 (blue)). The volume corresponds to a part of the somatosensory cortex of an adult mouse and was acquired using serial section electron microscopy (ssEM). The train and test volume dimensions are $85\times 1463\times 1613\,\text{ voxels }$ and $75\times 1334\times 1553\,\text{ voxels }$ respectively, with an anisotropic resolution of $3\times 3\times 30$ nm per voxel.

### Experimental Setup

**Evaluation metrics.** We evaluate our methods using the Jaccard index of the positive class or *foreground IoU*, defined as $IoU_{F} = TP/(TP+FP+FN)$ where TP are the true positives, FP the false positives and FN are the false negatives. As a convention, the positive class is foreground and the negative class is background. The background IoU is defined likewise by swapping the positive and negative classes. To obtain these values, the probability image returned by the network is binarized using a threshold value of 0.5. Nevertheless, to compare our results with other related works we also define the *overall IoU* as $IoU_{O} = (IoU_{F} + IoU_{B})/2$ where $IoU_{F}$ and $IoU_{B}$ are the foreground and background IoU, respectively. Notice the high proportion of background pixels typically inflates the overall IoU score, resulting in greater values than the foreground IoU.

**Training setup and data augmentation.** To find the best solutions, we made an exhaustive search of hyperparameters and training configurations, exploring different loss functions, optimizers, learning rates, batch sizes, and data augmentation techniques. We explored as well the use of different input patch sizes, their selection method (random or systematic), and the discarding of image patches with low foreground class information (Oztel et al., 2017). When selecting a random patch, we define a probability map to choose patches with a higher probability of containing mitochondria, therefore addressing the class imbalance problem. Finally, we have also studied the effect of selecting the validation set as either consecutive training images or at random. Here we describe the best training configuration found. However, the details of our exhaustive search are available in the supplementary material. In particular, for the 2D networks, we minimize the binary cross-entropy (BCE) loss using the Stochastic Gradient Descent (SGD) optimizer, 0.99 momentum and no decay, with a learning rate of 0.002, a batch size value of 6 and using a patch size $256\times 256$ pixels. The validation set is formed by $10\%$ of the training images selected at random. We use a GeForce GTX 1080 GPU card to train the network for 360 epochs, completing an epoch when all training data is explored, with a patience established at 100 epochs monitoring the validation loss and picking up the model that performs best in the validation set. Moreover, we apply on-the-fly data augmentation (DA) with random rotations and vertical and horizontal flips. For the 3D networks, the same hyperparameters as the 2D are used but we employ elastic transformations as well (in 2D we did not observe an improvement), using a patch size of $80\times 80\times 80$ voxels .

### Experiments on Lucchi Dataset

#### Reproducing Top State-Of-The-Art Methods

We aimed at reproducing the state-of-the-art deep learning-based methods that report top performance in the Lucchi dataset published by Cheng and Varshney (2017), Casser et al. (2020), Xiao et al. (2018) and Oztel et al. (2017). Only the code by Casser et al. (2020) is publicly available, so we plugged their network architecture into our training workflow. The code from the rest of the methods was unsuccessfully requested to their corresponding authors.

In all cases, a first implementation attempt was made following the methodology and exact parameters described in each publication. When finding missing information, we proceeded using the most common practice in the field. In addition, following the same procedure we use for our own models, we modified the original configuration (i.e., architecture and training workflow) aiming at improving the results and their stability (full details available in the supplementary material). These configurations are hereafter referred to as *original* and *modified* respectively. A systematic search of the best hyperparameters and training configurations was performed and the results are shown in Table 6.

**Table 1:** Foreground IoU (mean±standard deviation) of reproduced state-of-the-art works in Lucchi dataset. *Original* refers to the exact configurations as reported by the authors, while *Modified* corresponds to the best configuration found by us. The different output reconstruction and post-processing methods adopted are indicated. More details available in Table S3.1

				**50% Overlap**					**Full Image**		
**Network**	**Param. number**	**Reported**	**Per Patch**		**+Test-time aug.**	**+Test-time aug. + Z-Filtering**	**+ Blending +Test-time aug.**	**+Blending +Test-time aug. +Z. Filtering**		**+Test-time aug.**	**+Test-time aug. + Z-Filtering**
Cheng et. al (2D)	0.6M	0.865									
*Original*	0.59M		0.503±0.233	0.517±0.240	0.517±0.239	0.521±0.243	0.541±0.250	0.548±0.254	0.526±0.244	0.537±0.244	0.543±0.252
*Modified*	0.59M		0.848±0.012	0.851±0.011	0.863±0.010	0.868±0.010	0.865±0.008	0.871±0.008	0.853±0.011	0.865±0.009	0.871±0.008
Maximum	-		0.864	0.865	0.877	0.881	0.878	0.883	0.865	0.878	0.881
Casser et. al	1.96M	0.890									
*Original*	1.96M		0.824±0.014	0.815±0.016	0.825±0.013	0.831±0.013	0.831±0.011	0.838±0.011	0.820±0.016	0.833±0.011	0.839±0.012
*Modified*	1.96M		0.844±0.014	0.837±0.008	0.846±0.016	0.850±0.017	0.850±0.016	0.855±0.017	0.842±0.006	0.853±0.015	0.858±0.015
Maximum	-		0.846	0.846	0.861	0.865	0.862	0.867	0.848	0.865	0.870
Oztel et. al	0.14M	0.907									
*Original*	0.14M		-	-	-	-	-	-	0.425±0.080	0.457±0.060	0.466±0.061
*Modified*	0.07M			-	-	-	-	-	0.451±0.042	0.476±0.049	0.487±0.053
Maximum	-		-	-	-	-	-	-	0.500	0.531	0.544
Cheng et. al (3D)	0.63M	0.889									
*Original*	0.79M		0.053±0.000	0.053±0.000	0.053±0.000	0.053±0.000	-	-	-	-	-
*Modified*	0.79M		0.623±0.039	0.714±0.040	0.053±0.034	0.053±0.034	-	-	-	-	-
Maximum	-		0.694	0.787	0.799	0.800	-	-	-	-	-
Xiao et. al	1.1M	0.900									
*Original*	1.08M		0.874±0.003	0.863±0.004	0.866±0.004	0.867±0.004	-	-	-	-	-
*Modified*	1.08M		0.882±0.002	0.872±0.003	0.874±0.003	0.874±0.003	-	-	-	-	-
Maximum	-		0.885	0.880	0.880	0.880	-	-	-	-	-

The original 2D network configuration by Cheng and Varshney (2017) produces results with high standard deviation, probably due to the high learning rate employed (0.05), even though it is reduced when reaching the $50\%$ and $75\%$ of total epochs. Our modified configuration differs in the optimizer used (Adam instead of SGD) and learning rate (fixed to 0.0001). Additionally, we performed extra DA with random rotations, removed the dropout layers, reduced the number of epochs and extracted 12 random patches per training image instead of just one. Without post-processing (none is used in the original publication), the foreground IoU value reported (0.865) can only be reached through our modified configuration and by taking the maximum values of the $50\%$ *overlap* or *full image* reconstruction strategies. Even better values can be obtained thanks to post-processing. The 3D approach of the same authors, Cheng and Varshney (2017), produces IoU values close to 0 in its original form, since using the proposed learning rate (0.1), the network gets easily trapped in local minima. Moreover, the subvolume shape adopted, $128\times 128\times 96\ \text{ pixels }$, makes train/validation data splitting difficult, so we train the network until convergence with no validation data. Our modified configuration produces better results but far from the reported ones and highly unstable (0.800 in its best run vs the reported 0.889).

The original configuration proposed by Casser et al. (2020) reaches high IoU values with low standard deviation as well. We modified it by selecting two random patches per training image instead of one and using a probability map to prioritize patches having mitochondria pixels in the center, which leads to more stable results. The maximum value was obtained by applying Z-filtering to the predictions over full test images, measuring 0.870 of foreground IoU. In the original code, the authors optimized the training by using the test set as validation set, which could explain their better reported value.

The work presented by Xiao et al. (2018) provided a detailed explanation of their training procedure, architecture and output reconstruction strategy. Thus, the only modification we made is the use of elastic transformations in DA. As it is shown in Table 1, this change improves substantially the results obtained. They merge the predictions with overlap and ensemble, so to be fair, the maximum value of patch merging using $50\%$ overlap and ensemble predictions should be used for comparison. They reported 0.900 of foreground IoU compared to the maximum 0.880 achieved by our modified version.

Finally, the original configuration proposed by Oztel et al. (2017) produces very low foreground IoU values. We reproduced their model and tried modifying their network by adding more non-linearities (ReLU), changing the dropout values or the feature maps used, but the results obtained are far from those presented by the authors. The number of parameters in the original network compared with other state-of-the-art approaches is also relatively low (0.14M). Furthermore, we implemented their post-processing pipeline, whose results are presented in Table 2. We adapted it to specifically improve the segmentation made by the proposed network. Although the final metric value increased by a large margin, our results are far from their reported IoU.

The instructions to reproduce all models can be found at our official documentation site: https://em-image-segmentation.readthedocs.io/en/latest/manuscripts/stable_mitochondria.html. In addition, the details of each experiment can be found in the supplementary material, with a link to the template that reproduces its results.

**Table 2:** Foreground IoU results by the original and modified configurations of (Oztel et al., 2017) using their consecutive post-processing methods, i.e., *Spurious Detection* is applied over *Full Images*, then they are passed through *Watershed*, and finally through Z-filtering

	**Full Image**	**Spurious Detection**	**Watershed**	**Z-Filtering**
*Original*	0.425±0.080	0.426±0.091	0.540±0.100	0.573±0.106
*Modified*	0.451±0.042	0.449±0.067	0.562±0.057	0.599±0.067
Maximum	0.500	0.539	0.619	0.683

#### Proposed Networks Vs. State-Of-The-Art Networks for Semantic Segmentation

Here, we introduce the performance of our proposed architectures together with a study in-depth of the main state-of-the-art semantic segmentation networks for natural and biomedical images. Namely, FCN 8/32 (Long et al., 2015), MultiResUNet (Ibtehaz & Rahman, 2020), MNet (Fu et al., 2018), Tiramisu (Jégou et al., 2017), U-Net++ (Zhou et al., 2018), 3D Vanilla U-Net (Çiçek et al., 2016) and nnU-Net (Isensee et al., 2021). All implementations have been obtained or ported from their official sites and all networks have been optimized under the same conditions: same training and validation partitions, DA, optimizers and learning rate ranges (see supplementary material). The case of the nnU-Net is special since it is designed to optimize the whole segmentation pipeline. For a fair comparison, we extracted the optimal architecture found following the nnU-Net regular processing and plugged it into our own workflow.

**Table 3:** Performance of proposed and state-of-the-art networks for semantic segmentation in the Lucchi dataset (foreground IoU, mean±standard deviation). Scores are shown using the different post-processing and output reconstruction methods adopted. 3D patches required a minimum overlap so they are marked with *. Best results of each column and type of network (2D or 3D) are shown in bold. More details in Table S3.2

			**50% Overlap**					**Full Image**		
Network	Param. Number	Per Patch		*Test-time aug.	*Test-time aug. *Z-Filtering	*Blending *Test-time aug.	*Blending *Test-time aug. *Z-Filtering		*Test-time aug.	*Test-time aug. *Z-Filtering
FCN 32 (Dai et al., 2016)	50.38M	0.040±0.000	0.677±0.005	0.679±0.006	0.680±0.006	0.659±0.004	0.661±0.004	0.657±0.003	0.659±0.003	0.660±0.003
MultiResUNet (Ibtehaz & Rahman, 2020)	7.26M	0.815±0.000	0.814±0.014	0.820±0.010	0.824±0.010	0.834±0.010	0.840±0.009	0.828±0.016	0.833±0.010	0.839±0.010
Tiramisu (Jégou et al., 2017)	9.4M	0.810±0.028	0.833±0.027	0.851±0.018	0.857±0.017	0.850±0.016	0.855±0.016	0.830±0.029	0.846±0.019	0.851±0.018
MNet (Fu et al., 2018)	8.54M	0.851±0.011	0.865±0.008	0.870±0.007	0.874±0.007	0.874±0.006	0.878±0.006	0.867±0.008	0.872±0.006	0.876±0.008
U-Net++ (Zhou et al., 2018)	37.7M	0.734±0.012	0.872±0.005	0.877±0.004	0.881±0.004	**0.880**$\varvec{\pm }$**0.003**	0.884±0.003	**0.875**$\varvec{\pm }$**0.004**	**0.878**$\varvec{\pm }$**0.003**	**0.882**$\varvec{\pm }$**0.003**
2D SE U-Net (ours)	1.95M	0.863±0.002	0.873±0.003	0.878±0.003	0.882±0.003	0.880±0.003	0.883±0.003	0.875±0.002	0.881±0.002	0.881±0.002
2D Residual U-Net (ours)	2.03M	0.867±0.005	0.873±0.005	0.877±0.004	0.880±0.004	0.878±0.003	0.882±0.003	0.875±0.004	0.877±0.003	0.880±0.004
FCN 8 (Dai et al. 2016)	50.38M	0.860±0.005	0.880±0.003	0.884±0.002	0.888±0.002	**0.887**$\varvec{\pm }$**0.002**	0.891±0.002	0.881±0.003	0.886±0.002	**0.891**$\varvec{\pm }$**0.002**
nnU-Net (Isensee et al., 2021)	52.1M	0.867±0.004	0.876±0.004	0.881±0.003	0.884±0.003	0.882±0.003	0.886±0.003	0.861±0.007	0.864±0.009	0.868±0.008
2D U-Net (ours)	**1.95M**	0.874±0.003	0.881±0.002	0.884±0.002	0.888±0.002	0.884±0.000	0.889±0.002	0.882±0.003	0.884±0.002	0.887±0.003
2D Attention U-Net (ours)	1.99M	**0.875**$\varvec{\pm }$**0.004**	**0.882**$\varvec{\pm }$**0.003**	**0.885**$\varvec{\pm }$**0.001**	**0.890**$\varvec{\pm }$**0.002**	0.886±0.001	**0.892**$\varvec{\pm }$**0.001**	**0.884**$\varvec{\pm }$**0.002**	**0.886**$\varvec{\pm }$**0.001**	0.890±0.002
3D Vanilla U-Net (Çiçek et al., 2016)	19.07M	0.402±0.005(*)	0.851±0.004	0.857±0.006	0.857±0.006	-	-	-	-	-
3D SE U-Net (ours)	0.79M	0.387±0.007(*)	0.867±0.009	0.873±0.007	0.874±0.007	-	-	-	-	-
3D Attention U-Net (ours)	0.79M	0.389±0.005(*)	0.870±0.003	0.876±0.003	0.876±0.003	-	-	-	-	-
3D U-Net (ours)	**0.79M**	0.394±0.005(*)	0.871±0.006	0.878±0.004	0.878±0.004	-	-	-	-	-
3D Residual U-Net (ours)	1.50M	**0.394**$\varvec{\pm }$0.004*	**0.877**$\varvec{\pm }$**0.004**	**0.883**$\varvec{\pm }$**0.002**	**0.883**$\varvec{\pm }$**0.002**	-	-	-	-	-

All 2D networks use an input patch size of $256\times 256$ pixels, while 3D networks use $80\times 80\times 80\;\text{ voxels }$ subvolumes to exploit the isotropic resolution of the Lucchi dataset. The results from the best configuration found for each network are shown in Table 3. Notice the 3D networks do not have results using full image reconstructions due to GPU memory limitations, as the whole dataset should be fed to the network. Similarly, blending estimation was not implemented in 3D networks given their computational cost.

**Performance of state-of-the-art biomedical segmentation networks.** The results of Tiramisu (Jégou et al., 2017), MNet (Fu et al., 2018), nnU-Net (Isensee et al., 2021), MultiResUNet (Ibtehaz & Rahman, 2020) and 3D Vanilla U-Net (Çiçek et al., 2016) are below 0.880 of foreground IoU even when using output reconstructions with 50% of overlap and post-processing techniques such as ensemble predictions or Z-filtering. On top of these networks, the U-Net++ achieved the best results, scoring $0.881\pm 0.004$ of foreground IoU. The 3D Vanilla U-Net, nnU-Net, U-Net++ and MNet seem to produce stable results (low standard deviation), while Tiramisu and MultiResUNet have larger variability within their results. Besides that, the difference in their number of trainable parameters is remarkable. The 3D Vanilla U-Net, nnU-Net and U-Net++ models have between $2\times$ and $5\times$ more parameters than the other state-of-the-art approaches. Concerning the FCN networks (Long et al., 2015), the FCN32 reports low IoU values while the FCN8 achieves results comparable with our best 2D U-Net configuration. Nevertheless, the number of trainable parameters in FCN8 is 50.38M compared to less than 2M in our proposed 2D models.

**Performance of our proposed networks.** Regarding our proposed approaches (“Proposed networks”), the best values were obtained with the 2D U-Net and its version with attention gates: $0.888\pm 0.002$ and $0.890\pm 0.002$ applying test-time data augmentation and Z-filtering post-processing respectively. Our 3D networks do not reach the performance obtained with 2D versions. This may be explained by inspecting mitochondria labels in 3D, we observed they frequently lose shape continuity through slices, penalizing the learning capacity of 3D networks (see Fig. S2.2).

Remarkably, our 3D networks have three times fewer training parameters than our 2D approaches, leading to more computationally efficient models. To complete the overview of the state-of-the-art networks and architectures, we experimented with *Squeeze-and-Excitation* (SE) blocks (Hu et al., 2018) in our proposed 2D and 3D models. These blocks perform dynamic channel-wise feature recalibration by *squeeze* and *excite* operations. The *Squeeze* operation consists of collecting global spatial information into a channel descriptor using global average pooling. After that, features are recalibrated by the *excite* operation, which emphasizes channel-wise features with a simple gating mechanism based on a ReLU and a Sigmoid activation. Their best results are obtained with SE blocks everywhere except the bottleneck, as suggested by Roy et al. (2018). Nevertheless, we experimented as well with inserting SE blocks after every convolutional layer. As shown in Table 3, these blocks do not imply a boost in performance in this case.

A full description of the configurations tested can be found in Section S3 (supplementary material).

#### Comparison with Reported Results

We have summarized in Table 4 the reported results of the top-performing published methods, together with those of state-of-the-art approaches and our proposed networks. All reproduced values correspond to the best configuration found, i.e., using the optimal pre-processing, architecture, output reconstruction, and post-processing strategies for each method. The availability of original code, including that of the present paper, is also indicated. Notice the gap between the averaged IoU and the reported values increases with the standard deviation, underling the importance of finding stable configurations so as not to depend on a large computation budget (Dodge et al., 2019).

**Table 4:** Reported vs. reproduced scores in the Lucchi dataset. The *Reported* values correspond to the scores claimed by authors of each publication or the maximum score obtained by us. The *Reproduced* values refer to the maximum, mean and standard deviation obtained while reproducing each corresponding method. Best scores of each column are presented in bold

			**Foreground IoU**		**Overall IoU**	
**Description**	**Implementation**	**Code**	**Reported**	**Reproduced**	**Reported**	**Reproduced**
FCN 32	Ours using (Dai et al., 2016)	$\checkmark$	0.688	0.688 (0.680±0.006)	0.835	0.835 (0.831±0.003)
MultiResUNet	Ours using (Ibtehaz & Rahman, 2020)	$\checkmark$	0.847	0.847 (0.824±0.010)	0.919	0.919 (0.902±0.007)
2D CNN	(Cheng & Varshney, 2017)		0.865	0.883 (0.871±0.008)	-	0.938 (0.932±0.004)
3D Vanilla U-Net	Ours using (Çiçek et al., 2016)	$\checkmark$	0.866	0.866 (0.857±0.006)	0.929	0.929 (0.924±0.003)
Tiramisu	Ours using (Jégou et al., 2017)	$\checkmark$	0.872	0.872 (0.857±0.017)	0.932	0.932 (0.924±0.009)
2D U-Net	(Casser et al., 2020)	$\checkmark$	0.878	0.865 (0.853±0.015)	0.935	0.930 (0.922±0.007)
3D SE U-Net	Ours	$\checkmark$	0.879	0.879 (0.874±0.007)	0.936	0.936 (0.933±0.004)
3D Attention U-Net	Ours	$\checkmark$	0.880	0.880 (0.876±0.003)	0.936	0.936 (0.934±0.002)
nnU-Net framework	(Isensee et al., 2021)	$\checkmark$	0.882	-	0.938	-
MNet	Ours using (Fu et al., 2018)	$\checkmark$	0.883	0.883 (0.874±0.007)	0.938	0.938 (0.929±0.004)
2D Residual U-Net	Ours	$\checkmark$	0.885	0.885 (0.880±0.004)	0.939	0.939 (0.937±0.002)
3D U-Net	Ours	$\checkmark$	0.885	0.885 (0.878±0.004)	0.939	0.939 (0.935±0.002)
nnU-Net	Ours using (Isensee et al., 2021)	$\checkmark$	0.888	0.888 (0.881±0.005)	0.941	0.941 (0.937±0.003)
3D Residual U-Net	Ours	$\checkmark$	0.888	0.888 (0.883±0.002)	0.941	0.941 (0.938±0.001)
2D SE U-Net	Ours	$\checkmark$	0.888	0.888 (0.882±0.003)	0.941	0.941 (0.937±0.002)
U-Net++	Ours using (Zhou et al., 2018)	$\checkmark$	0.888	0.888 (0.884±0.003)	0.941	0.941 (0.938±0.001)
3D CNN	(Cheng & Varshney, 2017)		0.889	0.800 (0.738±0.034)	-	0.894 (0.860±0.018)
2D U-Net+Z-filtering	(Casser et al., 2020)	$\checkmark$	0.890	0.870 (0.858±0.015)	0.942	0.931 (0.925±0.007)
FCN 8	Ours using (Dai et al., 2016)	$\checkmark$	0.893	**0.893 (0.888±0.002)**	**0.943**	**0.943 (0.941±0.001)**
2D U-Net	Ours	$\checkmark$	0.893	**0.893 (0.888±0.002)**	0.942	0.942 (0.941±0.001)
2D Attention U-Net	Ours	$\checkmark$	0.893	**0.893 (0.890±0.002)**	**0.943**	**0.943 (0.942±0.001)**
3D U-Net	(Xiao et al., 2018)		0.900	0.881 (0.875±0.003)	-	0.937 (0.934±0.002)
CNN+3 Post-proc.	(Oztel et al., 2017)		**0.907**	0.683 (0.599±0.067)	-	0.800 (0.757±0.106)

Our proposed 2D U-Net and Attention U-Net models, together with the FCN8 model reached the highest reproducible foreground IoU score with a value of 0.893. In particular, the 2D Attention U-Net achieved a slightly higher average score in a very consistent manner. Best values were obtained using blending and ensemble for output reconstruction and Z-filtering as post-processing (see Fig. S1.1 for an example of some of the proposed networks’ predictions). As opposed to other approaches, the standard deviation of our results is consistently low, guaranteeing good performance and reducing the number of experiments needed to reach optimal segmentation.

As expected, the lack of code associated with a publication enormously hinders the reproduction of the claimed results. Interestingly, in the case of the 2D approach by Cheng and Varshney (2017), our implementation improved over their published results, stressing the benefits of optimizing the whole segmentation workflow. Notice there are two table entries for results with nnU-Net (Isensee et al., 2021): one using their entire training framework, and one plugging the best architecture found by their framework into ours.

#### Ablation Study

To investigate the relevance of each component in our proposed networks, we performed an ablation study of our 2D U-Net baseline architecture. We compared six ablated versions with incremental changes: 1) a baseline four-level 2D U-Net model containing ReLU activations, Glorot uniform kernel initialization (Glorot & Bengio, 2010), 16 feature maps in the first level of the network that are doubled on each level, and no regularization or DA; 2) the baseline with basic DA (random rotations and horizontal and vertical flips); 3) adding batch normalization 4) adding dropout as regularization method; 5) using ELU as activation function ($\alpha =1$); 6) using *He normal* (He et al., 2015) as kernel initialization; 7) adding attention gates (Schlemper et al., 2019) in the skip connections.

**Table 5:** Ablation study of our full 2D model. From the top to the bottom, on each row, incremental modifications are applied based on the previous configuration, except batch normalization, which was discarded as it decreases the performance

	**Foreground IoU**		
**Method**	**Per Patch**	**50% Overlap**	**Full Image**
Baseline - 2D U-Net	0.725±0.020	0.748±0.027	0.739±0.002
+ DA	0.859±0.007	0.872±0.003	0.871±0.004
(+ Batch norm.)	0.856±0.005	0.864±0.004	0.869±0.002
+ Dropout	0.870±0.003	0.880±0.002	0.881±0.002
+ ELU activation	0.873±0.003	0.880±0.001	0.881±0.002
+ He initializer	0.873±0.003	0.880±0.002	0.881±0.003
+ Attention Gates	**0.875±0.003**	**0.882±0.003**	**0.884±0.002**

The evaluation results on the Lucchi dataset for each case are shown in Table 5. Notice the IoU values vary significantly if they are provided by patch or by reconstructing the final output, highlighting once more the need of specifying the framework chosen when presenting the results. The use of DA together with dropout clearly outperforms the baseline architecture by a large margin. Batch normalization decreases the performance, so it was not included in successive models. In the same way, the usage of ELU improves over the use of ReLU activation functions. Conversely, changing the kernel initialization from Glorot uniform to He normal has marginal effects in the final result, so either can be used. Finally, introducing attention in the skip connections, as suggested by Schlemper et al. (2019), helped increasing the network performance and maintaining results stability.

A comprehensive study on how the different IoU values of the ablation results relate to the segmented size and shape of the reconstructed mitochondria is presented in Section S4 (supplementary material).

### Results on Lucchi++ and Kasthuri++

To test how well the best solutions found for Lucchi would generalize in other datasets, we applied the same configurations to Lucchi++ and Kasthuri++ and compared their performance with that reported by Casser et al. (2020). In Table 6, we can see our models outperform all previously reported results by a large margin. Since these datasets corrected the mitochondria label continuity through the slices, the best performance is obtained with 3D networks. This supports the hypothesis that the Lucchi dataset labeling inconsistencies hinder the learning capacity of the 3D networks, which are usually expected to perform better than 2D networks in such a context (Wolf et al., 2018). Moreover, the Kasthuri++ dataset is anisotropic (lower resolution in the z-axis). Therefore, we modified our proposed 3D networks by removing the z-axis downsampling in their pooling operations and using shallower architectures (three levels instead of four).

**Table 6:** Results obtained in the Lucchi++ and Kasthuri++ datasets. All our model scores correspond to optimal architectures found in Lucchi

			**Foreground IoU**		**Overall IoU**	
**Dataset**	**Description**	**Author**	**Maximum**	**(mean±std)**	**Maximum**	**(mean±std)**
**Lucchi++**	2D U-Net	Casser et al. (2020)	0.888	-	0.940	-
	2D U-Net+Z Filtering	Casser et al. (2020)	0.900	-	0.946	-
	2D Residual U-Net (*)	Ours	0.908	0.904±0.004	0.943	0.948±0.002
	2D U-Net (*)	Ours	0.916	0.911±0.006	0.955	0.952±0.003
	2D Attention U-Net (*)	Ours	0.919	0.914±0.003	0.956	0.954±0.001
	3D U-Net (a)	Ours	0.923	0.915±0.007	0.958	0.954±0.004
	3D Attention U-Net (a)	Ours	0.923	0.912±0.008	0.959	0.953±0.004
	3D Residual U-Net (a)	Ours	**0.926**	0.919±0.005	**0.960**	0.957±0.003
**Kasthuri++**	2D U-Net	Casser et al. (2020)	0.845	-	0.920	-
	2D U-Net+Z Filtering	Casser et al. (2020)	0.846	-	0.920	-
	2D Residual U-Net (a)	Ours	0.908	0.906±0.001	0.953	0.950±0.001
	2D Attention U-Net (a)	Ours	0.915	0.913±0.001	0.956	0.954±0.001
	2D U-Net (a)	Ours	0.916	0.913±0.002	0.955	0.954±0.001
	3D U-Net (a)	Ours	0.934	0.932±0.001	0.965	0.965±0.001
	3D Residual U-Net (a)	Ours	0.934	0.933±0.001	0.966	0.966±0.000
	3D Attention U-Net (a)	Ours	**0.937**	0.934±0.001	**0.967**	0.966±0.001

(*) 0% overlap output reconstruction, blended ensemble and z-filtering post-processing

(a) 50% overlap output reconstruction and ensemble post-processing

## Conclusion

By a complete experimental study of state-of-the-art DL models with modern training workflows, we have revealed significant problems of reproducibility in the domain of mitochondria segmentation in EM data. Moreover, by disentangling the effects of novel architectures from those of the training choices (i.e., pre-processing, data augmentation, output reconstruction, and post-processing strategies) over a set of multiple executions of the same configurations, we have found stable lightweight models that consistently lead to state-of-the-art results on the existing public datasets.

Have novel methods reached human performance? To answer that question, Casser et al. (2020) compared the results of human annotators in the Lucchi dataset, producing a foreground IoU value of 0.884. This would suggest that many of the models presented in Table 4 outperform indeed humans in this task. Nevertheless, all methods fall short of the 0.907 threshold for foreground IoU red reported by Oztel et al. (2017), which could be due to the annotation inconsistencies discussed in “Proposed networks vs. state-of-the-art networks for semantic segmentation”. To investigate further, we created two slightly different versions of the mitochondria ground truth labels by 1-pixel morphological dilation and erosion. The foreground IoU value of the resulting labels against the original ground truth was 0.885 (dilatation) and 0.904 (erosion). Thus, this enforces the idea that the dataset is not pixel-level accurate, so it could be argued that all the methods with IoU values within a range of 0.009 or less can be considered to have similar performance. The same experiment was done with the ground truth labels of Lucchi++ (foreground IoU: 0.898, 0.919) and Kasthuri++ (foreground IoU: 0.927, 0.922). Indeed, even the average score of many of our models outperform those values (Table 6). This suggests the performance on all three datasets has probably saturated, as new architectures and training frameworks cannot improve beyond the limits inherent to semantic segmentation and the size of the datasets.

In closing, we believe further progress in mitochondria segmentation in EM will require (1) larger and more complex datasets (Wei et al., 2020), and (2) the adoption of a reproducibility checklist or set of best practices (Dodge et al., 2019) to report more comprehensive results and allow robust future comparisons.

## Information Sharing Statement

The datasets utilized for the training and testing of the models presented in this work are freely available.

## Supplementary Information

Below is the link to the electronic supplementary material.

Supplementary file1 (PDF 8.19 MB)

## Data Availability

The developed software that support the findings of this study are publicly available from Github https://github.com/danifranco/EM_Image_Segmentation.

## Data Availability

Lucchi dataset is available at https://www.epfl.ch/labs/cvlab/data/data-em/. Lucchi++ and Kasthuri++ datasets can be downloaded from https://sites.google.com/view/connectomics/.
