# Supplementary material for: Stable Deep Neural Network Architectures for Mitochondria Segmentation on Electron Microscopy Volumes
Source: Neuroinformatics. 2021 Dec 2;20(2):437–50. doi: 10.1007/s12021-021-09556-1 (PMC9546980; doi:10.1007/s12021-021-09556-1)
Supplement: Supplementary file 1 — Supplementary file1 (PDF 8.19 MB) [file 12021_2021_9556_MOESM1_ESM.pdf]

Supplementary material
Stable deep neural network architectures for mitochondria segmentation on electron microscopy volumes

Daniel Franco-Barranco[1,2], Arrate Muñoz-Barrutia[3,4], and Ignacio Arganda-Carreras[1,2,5]

[1] University of the Basque Country (UPV/EHU)
[2] Donostia International Physics Center (DIPC)
[3] Universidad Carlos III de Madrid
[4] Instituto de Investigación Sanitaria Gregorio Marañón
[5] Ikerbasque, Basque Foundation for Science
daniel.franco@dipc.org

S1 Network predictions on Lucchi

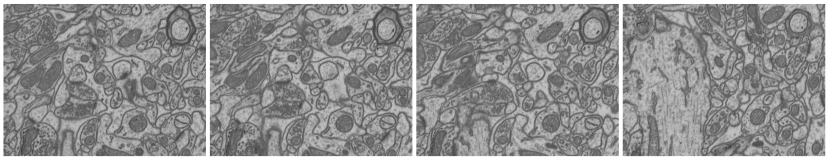

(a) Original images

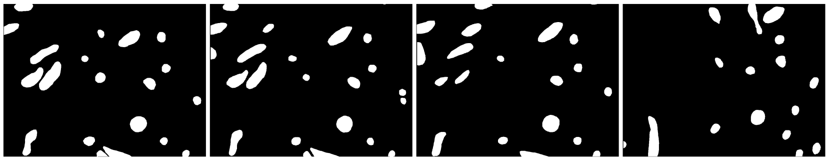

(b) Ground Truth

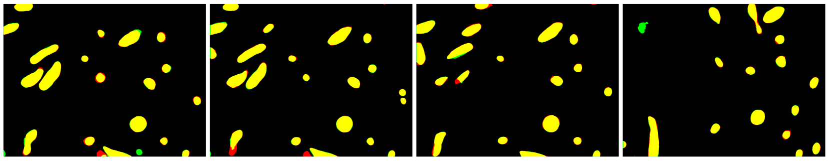

(c) Basic 2D U-Net predictions

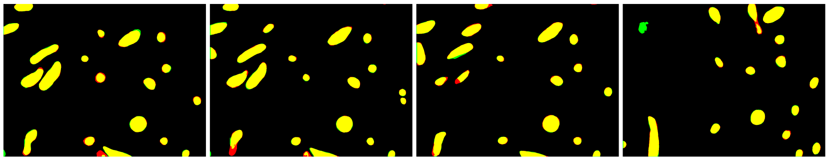

(d) 2D Attention U-Net predictions

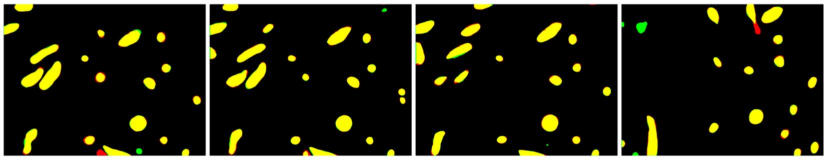

(e) 2D Residual U-Net predictions

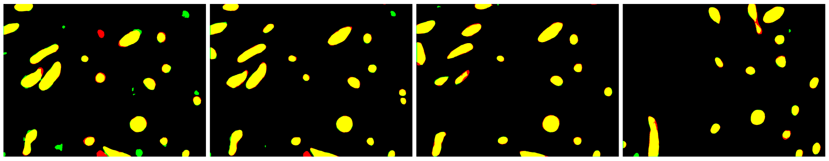

(f) Basic 3D U-Net predictions

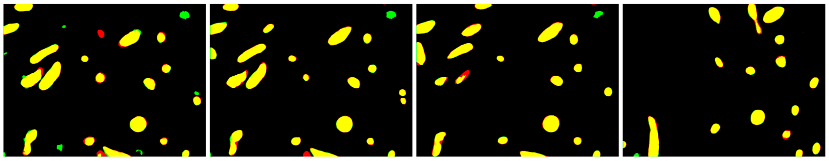

(g) 3D Attention U-Net predictions

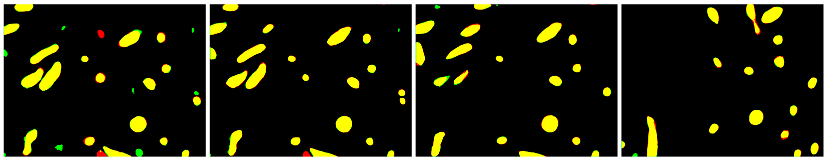

(h) 3D Residual U-Net predictions

Fig. S1.1: Predictions of the configuration that measures the best IoU score for each proposed network on Lucchi dataset. Yellow: True Positive; Green: False Positive; Red: False Negative.

S2 Lucchi and Lucchi++ label comparison

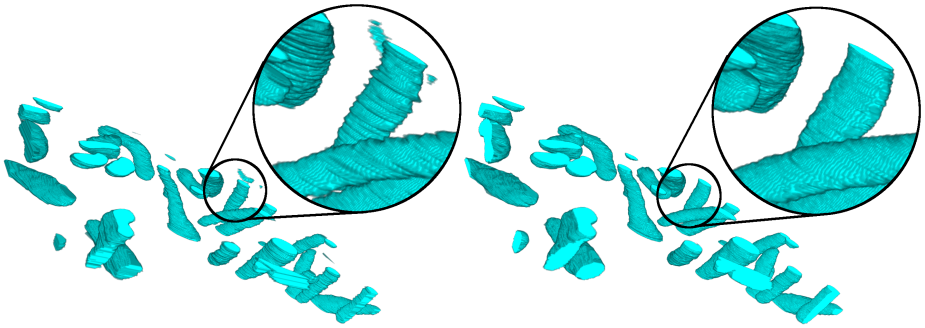

(a) (b)

Fig. S2.2: 3D views of mitochondria labels on Lucchi (a) and Lucchi++ (b).

S3 Fully detailed result tables

Network	Param. number	Reported	Per Patch	Per Image (no overlap)		Per image (50% overlap)					Full Image		
					+Z-Filtering		+Test-time aug.	+Test-time aug. +Z-Filtering	+Blending +Test-time aug.	+Blending +Test-time aug. +Z-Filtering		+Test-time aug.	+Test-time aug. +Z-Filtering
Cheng 2D [1]	0.6M	0.865											
Original	0.59M		0.503±0.233	0.503±0.233	0.511±0.238	0.517±0.240	0.517±0.239	0.521±0.243	0.541±0.250	0.548±0.254	0.526±0.244	0.537±0.244	0.543±0.252
Modified	0.59M		0.848±0.012	0.843±0.012	0.852±0.011	0.851±0.011	0.863±0.010	0.868±0.010	0.865±0.008	0.871±0.008	0.853±0.011	0.865±0.009	0.871±0.008
Maximum	-		0.864	0.858	0.865	0.865	0.877	0.881	0.878	0.883	0.865	0.878	0.881
Casser [2]	1.96M	0.890											
Original	1.96M		0.824±0.014	0.817±0.016	0.828±0.016	0.815±0.016	0.825±0.013	0.831±0.013	0.831±0.011	0.838±0.011	0.820±0.016	0.833±0.011	0.839±0.012
Modified	1.96M		0.844±0.014	0.838±0.008	0.845±0.009	0.837±0.008	0.846±0.016	0.850±0.017	0.850±0.016	0.855±0.017	0.842±0.006	0.853±0.015	0.858±0.015
Maximum	-		0.846	0.844	0.852	0.846	0.861	0.865	0.862	0.867	0.848	0.865	0.870
Oztel [3]	0.14M	0.907											
Original	0.14M		-	-	-	-	-	-	-	-	0.425±0.080	0.457±0.060	0.466±0.061
Modified	0.07M			-	-	-	-	-	-	-	0.451±0.042	0.476±0.049	0.487±0.053
Maximum	-		-	-	-	-	-	-	-	-	0.500	0.531	0.544
Cheng 3D [1]	0.63M	0.889											
Original	0.79M		0.053±0.000(†)	0.053±0.000(†)	0.053±0.000(†)	0.053±0.000	0.053±0.000	0.053±0.000	-	-	-	-	-
Modified	0.79M		0.623±0.039(†)	0.691±0.049(†)	0.693±0.049(†)	0.714±0.040	0.0737±0.034	0.738±0.034	-	-	-	-	-
Maximum	-		0.694	0.777	0.779	0.787	0.799	0.800	-	-	-	-	-
Xiao [4]	1.1M	0.900											
Original	1.08M		0.874±0.003(†)	0.863±0.004(†)	0.864±0.004(†)	0.863±0.004	0.866±0.004	0.867±0.004	-	-	-	-	-
Modified	1.08M		0.882±0.002(†)	0.873±0.003(†)	0.874±0.003(†)	0.872±0.003	0.874±0.003	0.874±0.003	-	-	-	-	-
Maximum	-		0.885	0.879	0.880	0.880	0.880	0.880	-	-	-	-	-

Table S3.1: Foreground IoU (mean±standard deviation) of reproduced state-of-the-art works in Lucchi dataset. This table is an extension of Table 1 of the main manuscript including an extra column for 'per image' reconstruction with no overlap. Different scores discussed in previous sections are shown, the post-processing methods adopted are indicated. *Original* versions refer to exact configurations as reported by the authors. *Modified* corresponds to our best approach modifying *Original* in some way to improve method's performance and results stability. The patch size and overlap (marked with †) in each work is as follows: 256×256 pixels for Cheng 2D, $128 \times 128 \times 96$ voxels ($0 \times 0 \times 27$ voxels overlap in $x \times y \times z$) for the subvolumes in Cheng 3D; 512×512 pixels (256×0 pixels overlap in $x \times y$) in Casser; $448 \times 576 \times 20$ voxels ($128 \times 128 \times 10$ voxels overlap in $x \times y \times z$) in Xiao; and 768×1024 pixels in Oztel.

Network	Param. number	Per Patch	Per image (no overlap)		Per image (50% overlap)					Full Image		
				+Z-Filtering		+Test-time aug.	+Test-time aug. +Z-Filtering	+Blending +Test-time aug.	+Blending +Test-time aug. +Z-Filtering		+Test-time aug.	+Test-time aug. +Z-Filtering
FCN 32 [5]	50.38M	0.040±0.000	0.637±0.005	0.640±0.005	0.677±0.005	0.679±0.006	0.680±0.006	0.659±0.004	0.661±0.004	0.657±0.003	0.659±0.003	0.660±0.003
MultiResUNet [6]	7.26M	0.815±0.000	0.812±0.016	0.821±0.015	0.814±0.014	0.820±0.010	0.824±0.010	0.834±0.010	0.840±0.009	0.828±0.016	0.833±0.010	0.839±0.010
Tiramisu [7]	9.4M	0.810±0.028	0.809±0.030	0.821±0.029	0.833±0.027	0.851±0.018	0.857±0.017	0.850±0.016	0.855±0.016	0.830±0.029	0.846±0.019	0.851±0.018
MNet [8]	8.54M	0.851±0.011	0.854±0.009	0.861±0.009	0.865±0.008	0.870±0.007	0.874±0.007	0.874±0.006	0.878±0.006	0.867±0.008	0.872±0.006	0.876±0.008
nnU-Net [9]	52.1M	0.845±0.009	0.845±0.009	0.853±0.010	0.854±0.011	0.872±0.005	0.876±0.006	0.876±0.006	0.881±0.005	0.799±0.052	0.788±0.066	0.790±0.068
U-Net++ [10]	37.7M	0.731±0.014	0.860±0.008	0.867±0.008	0.872±0.005	0.877±0.004	0.881±0.004	0.880±0.003	0.884±0.003	0.875±0.004	0.878±0.003	0.882±0.003
2D Residual U-Net (ours)	2.03M	0.867±0.005	0.864±0.005	0.871±0.006	0.873±0.005	0.877±0.004	0.880±0.004	0.878±0.003	0.882±0.003	0.875±0.004	0.877±0.003	0.880±0.004
2D SE U-Net (ours)	1.95M	0.863±0.002	0.861±0.003	0.869±0.003	0.873±0.003	0.878±0.003	0.882±0.003	0.880±0.003	0.883±0.003	0.875±0.002	0.881±0.002	0.881±0.002
FCN 8 [5]	50.38M	0.860±0.005	0.864±0.005	0.871±0.005	0.880±0.003	0.884±0.002	0.888±0.002	**0.887±0.002**	0.891±0.002	0.881±0.003	0.886±0.002	**0.891±0.002**
2D U-Net (ours)	**1.95M**	0.874±0.003	0.872±0.003	0.880±0.003	0.881±0.002	0.884±0.002	0.888±0.002	0.884±0.000	0.889±0.002	0.882±0.003	0.884±0.002	0.887±0.003
2D Attention U-Net (ours)	1.99M	**0.875±0.004**	**0.873±0.003**	**0.882±0.003**	**0.882±0.003**	**0.885±0.001**	**0.890±0.002**	0.886±0.001	**0.892±0.001**	**0.884±0.002**	**0.886±0.001**	0.890±0.002
3D Vanilla U-Net [11]	19.07M	0.402±0.005(†)	0.842±0.004(†)	0.844±0.005(†)	0.851±0.004	0.857±0.006	0.857±0.006	-	-	-	-	-
3D SE U-Net (ours)	0.79M	0.387±0.007(†)	0.854±0.013(†)	0.855±0.013(†)	0.867±0.009	0.873±0.007	0.874±0.007	-	-	-	-	-
3D Attention U-Net (ours)	0.79M	0.389±0.005(†)	0.856±0.003(†)	0.857±0.003(†)	0.870±0.003	0.876±0.003	0.876±0.003	-	-	-	-	-
3D U-Net (ours)	**0.79M**	0.394±0.005(†)	**0.858±0.007**(†)	**0.859±0.007**(†)	0.871±0.006	0.878±0.004	0.878±0.004	-	-	-	-	-
3D Residual U-Net (ours)	1.50M	**0.394±0.004**(†)	0.857±0.004(†)	0.858±0.004(†)	**0.877±0.004**	**0.883±0.002**	**0.883±0.002**	-	-	-	-	-

Table S3.2: Performance of proposed networks and state-of-the-art networks for semantic segmentation in the Lucchi dataset. This table is an extension of Table 2 of the main manuscript including an extra column for the 'per image' reconstruction with no overlap. All values represent the foreground IoU (mean±standard deviation). Scores are shown using the different post-processing methods adopted. In 3D patches a minimum overlap was required so they are marked with †. Best results of each column and type of network (2D or 3D) are shown in bold.

S4 Analysis of the impact of IoU on the reconstructed mitochondria morphology

To analyze how different IoU values modify the structures of the mitochondria segmented, we present in Figure S4.3 different predictions produced by experiments done in the ablation study with our proposed U-Net models.

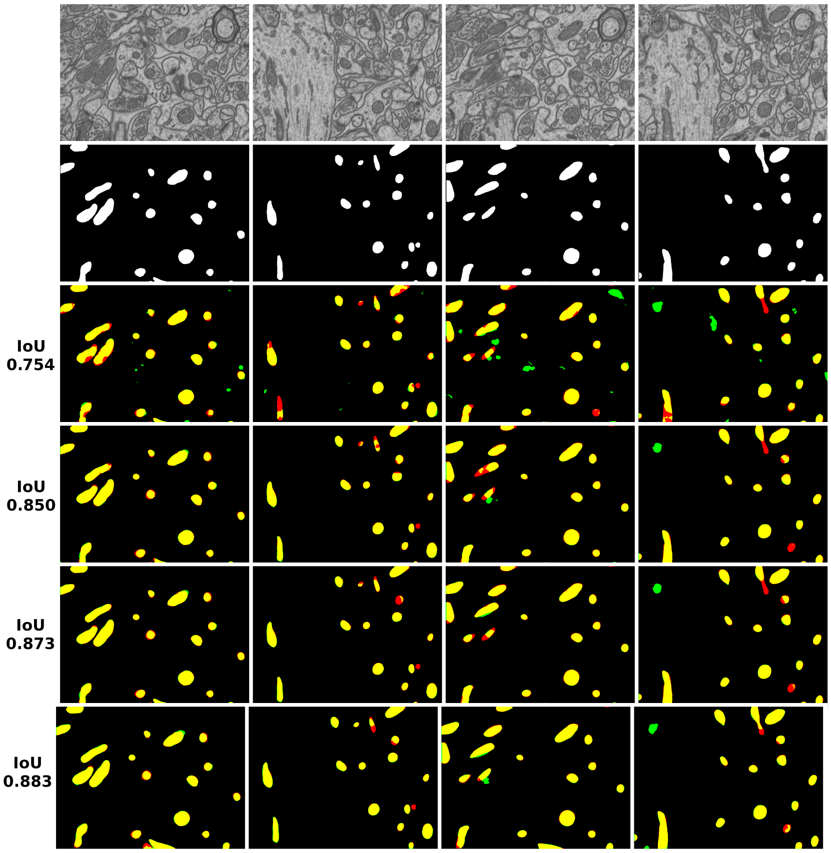

Fig. S4.3: Predictions from the ablation study of our proposed U-Net varying IoU performance. First and second row are original EM images and their ground truth masks respectively. Yellow: True Positive; Green: False Positive; Red: False Negative. Zoom the image to see better the differences between different IoU results.

Additionally, we measured the average volume, surface area and sphericity of the individual reconstructed mitochondria to quantify their size and characterize their shapes using MorphoLibJ [12]. To analyze full mitochondria only, tiny segments (with size under 3000 voxels) and segments touching the border of the images were discarded. As shown in Table S4.3, high IoU values correlate with a better approximation of the ground truth values for such measurements, proving IoU can be used as an indicator of final reconstruction performance.

More information about MorphoLibJ and the calculation of these metrics can be found in `https://imagej.net/plugins/morpholibj#measurements`.

**IoU**	**Instance number**	**Volume** (nm^3)	**SurfaceArea** (nm^2)	**Sphericity**
**0.754**	50	$9.57 \times 10^6 \pm 15.64 \times 10^6$	$341.16 \times 10^3 \pm 352.00 \times 10^3$	0.13 ± 0.10
**0.850**	27	$14.08 \times 10^6 \pm 17.78 \times 10^6$	$403.30 \times 10^3 \pm 321.32 \times 10^3$	0.24 ± 0.16
**0.873**	23	$17.11 \times 10^6 \pm 18.98 \times 10^6$	$437.29 \times 10^3 \pm 321.79 \times 10^3$	0.28 ± 0.15
**0.883**	22	$18.93 \times 10^6 \pm 19.91 \times 10^6$	$461.73 \times 10^3 \pm 336.46 \times 10^3$	0.27 ± 0.12
**1 (GT)**	20	$21.44 \times 10^6 \pm 20.00 \times 10^6$	$615.71 \times 10^3 \pm 416.43 \times 10^3$	0.20 ± 0.09

Table S4.3: Size and shape measurements (mean±standard deviation) on fully reconstructed mitochondria instances for different IoU values. Measurements were calculated with MorphoLibJ [12].

S5 Hyperparameter search space for each configuration

S5.1 Notations

- $[a, b]$: Range between two possible values. E.g. *zoom([0.75,1.25])* correspond to random zoom value between 0.75 and 1.25.
- $[a, b, c]$: All values from a to b with c step. E.g. $[10, 300, 10]$ correspond to $10, 20, 30, 40, ..., 300$.
- $choice[a, b, ...]$: one value between a, b and so on. E.g. *[10, 15, 20, 30, 60]* possible values are: 10 or 15 or 20 or 30 or 60 (but only one).
- $a, b, c, ...$: all tested values. E.g. flips, rotations, etc.

S5.2 Training setup

Computing Infrastructure	GeForce GTX 1080 Ti
GPU libraries	CUDA (10.1) + cuDNN (7.6.5)
Operating System	Ubuntu 16.04.6 LTS
Number of runs	10
Implementation	Tensorflow (2.1.0) + Keras (2.2.4-tf)
Code	Github
Math seed used	42
Environment used	Anaconda → DL_EM_base_env

S5.3 2D U-Net

Template to reproduce the results: U-Net_2D_template.py

Hyperparameter	Search space	Best assignment
Duplicate train	2	-
Validation	True	True
Random validation	True	True
% of train as validation	10%	10%
Patches	*choice*[random during train, created from train data before network training]	created from train data before network training
Patch size	256×256	256×256
Discard patches with less than a % of the foreground class	*choice*[True(5%), True(10%), True(15%), True(20%), True(30%), False]	False
Shuffle train on each epoch	*choice*[True,False]	True
Probability map	*choice*[False,True]	False
Probability for each class	*choice*[Foreground: 0.94 ; Background: 0.06, Foreground: 0.9 ; Background: 0.1]	-
Data augmentation	flips, rotation_range([-180,180]), square_rotations([0,90,180,270]), shearing([0.1,0.3]), shift([0.1,0.3]), brightness_range([0.8,1.2]), median_filtering(*choice*[1,3,5]), elastic, zoom([0.75,1.25])	flips, rotation_range([-180,180])
Number of epochs	[10,600,10]	360
Patience	choice [30,50,100]	50
Batch size	*choice*[3,5,6,9] and [1,64,×2]	6
Loss type	*choice*[BCE,Dice,Jaccard]	BCE
Optimizer	*choice*[SGD,Adam,Adabound]	SGD
SGD learning rate	*choice*[0.0001,0.0005,0.001, 0.002,0.005,0.01,0.05,0.1]	0.002
Adam learning rate	0.0001	-
Adabound learning rate	*choice*[(lr=0.0005,final_lr=0.1), (lr=0.0001,final_lr=0.1), (lr=0.001,final_lr=0.1), (lr=0.003,final_lr=0.1)]	-
Number of feature maps to start with (x2 and /2 of each down and up levels respec.)	*choice*[16,32,64]	16
Batch normalization	*choice*[True,False]	False
Dropout type	*choice*[dropout,spatial dropout]	dropout
Dropout	[0,0.4,0.1] and tiered on each downsampling: 0.1,0.2,0.3	tiered: 0.1,0.2,0.3
Pooling type	*choice*[Max-pooling,Average-pooling]	Max-pooling
Kernel initializer	*choice*[glorot_uniform,he_init]	he_init
Activation	*choice*[ReLU,ELU]	ELU

Table S5.4: Hyperparameter search space for the proposed 2D U-Net.

S5.4 2D Residual U-Net

Template to reproduce the results: Residual_U-Net_2D_template.py

**Hyperparameter**	**Search space**	**Best assignment**
Validation	True	True
Random validation	True	True
% of train as validation	10%	10%
Patches	created from train data before network training	created from train data before network training
Patch size	256×256	256×256
Data augmentation	flips, rotation_range([-180,180])	flips, rotation_range([-180,180])
Number of epochs	360	360
Patience	50	50
Batch size	*choice*[2,4,6,8]	6
Loss type	BCE	BCE
Optimizer	SGD	SGD
SGD learning rate	*choice*[0.001,0.002,0.003,0.004, 0.0001,0.0005,0.0007,0.0009]	0.002
Number of feature maps to start with (x2 and /2 of each down and up levels respec.)	*choice*[16,32]	16

Table S5.5: Hyperparameter search space for the proposed 2D Residual U-Net.

S5.5 3D U-Net

Template to reproduce the results: U-Net_3D_template.py

**Hyperparameter**	**Search space**	**Best assignment**
Validation	True	True
Random validation	*choice*[False, True]	False
% of train as validation	10%	10%
Subvolumes	created from train data before network training	created from train data before network training
Subvolume shape	*choice*[$80 \times 80 \times 80$, $128 \times 128 \times 48$, $128 \times 128 \times 80$, $128 \times 128 \times 128$, $112 \times 112 \times 112$]	$80 \times 80 \times 80$
Data augmentation	flips, square_rotations([0,90,180,270]), elastic, histogram_equalization, gaussian_blur(*choice*[$\sigma = (0, 2)$, $\sigma = (1, 2)$]), gamma_contrast(*choice*[$\sigma = (0.5, 2)$, $\sigma = (1.25, 1.75)$])	flips, elastic, square_rotations([0,90,180,270])
Number of epochs	360	360
Patience	200	200
Batch size	*choice*[1,2,4,6,8]	1
Loss type	BCE	BCE
Optimizer	*choice*[SGD,Adam]	Adam
Adam learning rate	*choice*[0.00001,0.00005,0.0001,0.0002, 0.0005,0.0007,0.001,0.005,0.01]	0.0001
SGD learning rate	*choice*[0.001]	-
Number of feature maps to start with (x2 and /2 of each down and up levels respec.)	*choice*[16,20,24,32]	-
Manually feature maps (on each level until bottleneck)	*choice*[{28,36,48,64,80,96}, {28,36,48,64,80}, {28,36,48,64}]	{28,36,48,64}
Dropout	*choice*[0,0.1,0.2]	-
Dropout type	*choice*[dropout,spatial_dropout]	-
Batch normalization	*choice*[False,True]	False
Network depth	*choice*[3,4,5]	3

Table S5.6: Hyperparameter search space for proposed 3D U-Net.

S5.6 3D Residual U-Net

Template to reproduce the results: Residual_U-Net_3D_template.py

**Hyperparameter**	**Search space**	**Best assignment**
Validation	True	True
Random validation	False	False
% of train as validation	10%	10%
Subvolumes	created from train data before network training	created from train data before network training
Subvolume shape	$80 \times 80 \times 80$	$80 \times 80 \times 80$
Data augmentation	flips, elastic, square_rotations([0,90,180,270])	flips, elastic, square_rotations([0,90,180,270])
Number of epochs	360	360
Patience	200	200
Batch size	*choice*[1,2,4,6]	1
Loss type	BCE	BCE
Optimizer	Adam	Adam
Adam learning rate	0.0001	0.0001
Number of feature maps to start with (x2 and /2 of each down and up levels respec.)	*choice*[16,32]	-
Manually feature maps (on each level until bottleneck)	*choice*[{28,36,48,64,80,96}, {28,36,48,64,80}, {28,36,48,64}]	{28,36,48,64,80}
Dropout	*choice*[0,0.1]	-
Dropout type	*choice*[dropout,spatial_dropout]	-
Batch normalization	*choice*[False,True]	False
Network depth	*choice*[3,4,5]	4

Table S5.7: Hyperparameter search space for proposed 3D Residual U-Net.

S5.7 Cheng 2D [1]

Templates to reproduce the results:
Original: cheng_2D_template_V0.py
Modified: cheng_2D_template_V1.py

Hyperparameter	Search space	Best assignment
Duplicate train	*choice*[1,12]	12
Validation	True	True
Random validation	True	True
% of train as validation	10%	10%
Patches	random selection from the whole data during train	random selection from the whole data during train
Patch shape	256×256	256×256
Probability map	*choice*[False,True]	True
Probability for each class	Foreground: 0.94 ; Background: 0.06	Foreground: 0.94 ; Background: 0.06
Data augmentation	flips, rotation_range([-180,180])	flips, rotation_range([-180,180])
Number of epochs	*choice*[4000,400]	400
Patience	200	200
Batch size	24	24
Loss type	BCE	BCE
Optimizer	*choice*[SGD,Adam]	Adam
SGD learning rate	*choice*[0.002,0.05]	-
Adam learning rate	*choice*[0.0001]	0.0001
learning rate scheduler	*choice*[True,False]	False
Dropout	*choice*[0,0.1]	0

Table S5.8: Hyperparameter search space for 2D network proposed by Cheng *et al.* [1].

S5.8 Cheng 3D [1]

Templates to reproduce the results:
***Original*:** cheng_3D_template_V0.py
***Modified*:** cheng_3D_template_V1.py

**Hyperparameter**	**Search space**	**Best assignment**
Duplicate train	*choice*[1,12]	12
Validation	False	False
Subvolumes	*choice*[created from train data before network training, random selection from the whole data during training]	created from training data before network training
Subvolume shape	$128 \times 128 \times 96$	$128 \times 128 \times 96$
Probability map	*choice*[False,True]	False
Data augmentation	flips, square_rotations([0,90,180,270]), elastic	square_rotations([0,90,180,270])
Number of epochs	*choice*[545,150]	150
Patience	*choice*[50,200]	50
Batch size	*choice*[1,3]	3
Loss type	BCE	BCE
Optimizer	*choice*[SGD,Adam]	Adam
SGD learning rate	0.1	-
Adam learning rate	0.0001	0.0001
learning rate scheduler	*choice*[True,False]	False
Dropout	0.1	0.1

Table S5.9: Hyperparameter search space for 3D network proposed by Cheng *et al.* [1].

S5.9 Casser [2]

Templates to reproduce the results:
***Original*:** casser_template_V0.py
***Modified*:** casser_template_V1.py

**Hyperparameter**	**Search space**	**Best assignment**
Duplicate train	*choice*[1,2,12]	2
Validation	True	True
Random validation	*choice*[True,False]	False
% of train as validation	*choice*[5%,10%,20%,30%]	10%
Patches	random selection from the whole data during train	random selection from the whole data during train
Patch size	512×512	512×512
Probability map	*choice*[False,True]	True
Probability for each class	Foreground: 0.9 ; Background: 0.1, Foreground: 0.94 ; Background: 0.06,	Foreground: 0.94 ; Background: 0.06
Data augmentation	flips, square_rotations([0,90,180,270]), rotation_range([0,180]), shift([0.1,0.3]), shearing([0.1,0.3]), brightness_range([0.8,1.2]), median_filtering(size=5)	flips, rotation_range([0,180])
Number of epochs	360	360
Patience	*choice*[50,200]	200
Batch size	*choice*[4,6]	4
Loss type	BCE	BCE
Optimizer	*choice*[SGD, Adam]	Adam
SGD learning rate	*choice*[0.001,0.002,0.005,0.008,0.01]	-
Adam learning rate	*choice*[0.0005,0.0001,0.001]	0.0005
Dropout	0.2	0.2

Table S5.10: Hyperparameter search space for network proposed by Casser *et al.* [2].

S5.10 Xiao [4]

Templates to reproduce the results:
Original: xiao_template_V0.py
Modified: xiao_template_V1.py

**Hyperparameter**	**Search space**	**Best assignment**
Duplicate train	*choice*[70,75,80,85]	75
Validation	True	False
Random validation	*choice*[True,False]	True
% of train as validation	*choice*[10%,20%]	10%
Subvolumes	created from train data before network training	created from train data before network training
Subvolume shape	$256 \times 256 \times 20$ (train) $448 \times 576 \times 20$ (test)	$256 \times 256 \times 20$ (train) $448 \times 576 \times 20$ (test)
Data augmentation	flips, square_rotations([0,90,180,270]), elastic	flips, square_rotations([0,90,180,270]), elastic
Number of epochs	30	30
Patience	30	30
Batch size	2	2
Loss type	BCE	BCE
Optimizer	Adam	Adam
Adam learning rate	0.0001	0.0001
Last network layer	*choice*[sigmoid,softmax]	softmax
L2 normalization	*choice*[0.1,0.01,0.001]	0.01

Table S5.11: Hyperparameter search space for network proposed by Xiao *et al.* [4].

S5.11 Oztel [3]

Templates to reproduce the results:
***Original*:** oztel_template_V0.py
***Modified*:** oztel_template_V1.py

Hyperparameter	Search space	Best assignment
Duplicate mitochondria samples	*choice*[2,3,6]	2
Reduce background samples	preserve 78% of samples	-
Validation	True	True
Random validation	True	True
% of train as validation	*choice*[10%,20%]	20%
Patches	created from train data before network training	created from train data before network training
Patch size	32×32	32×32
Data augmentation	flips, rotation_range([-180,180])	flips, rotation_range([-180,180])
Number of epochs	360	360
Patience	360	360
Batch size	32	32
Loss type	*choice*[CCE,BCE]	CCE
Optimizer	Adam	Adam
Adam learning rate	0.0001	0.0001

Table S5.12: Hyperparameter search space for network proposed by Oztel *et al.* [3].

S5.12 FCN [13]

Templates to reproduce the results:
FCN32: FCN32_template.py
FCN8: FCN8_template.py

Hyperparameter	Search space	Best assignment
Validation	True	True
Random validation	True	True
% of train as validation	10%	10%
Patches	created from train data before network training	created from train data before network training
Patch size	256×256	256×256
Data augmentation	flips, rotation_range([-180,180])	flips, rotation_range([-180,180])
Number of epochs	360	360
Patience	200	200
Batch size	6	6
Loss type	BCE	BCE
Optimizer	*choice*[SGD,Adam]	Adam
SGD learning rate	0.002	-
Adam learning rate	0.0001	0.0001

Table S5.13: Hyperparameter search space for FCN32 anf FCN8 networks [13].

S5.13 Tiramisu [7]

Template to reproduce the results: Tiramisu_template.py

**Hyperparameter**	**Search space**	**Best assignment**
Validation	True	True
Random validation	True	True
% of train as validation	10%	10%
Patches	created from train data before network training	created from train data before network training
Patch size	256 × 256	256 × 256
Data augmentation	flips, rotation_range([-180,180])	flips, rotation_range([-180,180])
Number of epochs	360	360
Patience	200	200
Batch size	*choice*[1,2,4,6,8,16]	4
Loss type	BCE	BCE
Optimizer	*choice*[SGD,Adam]	Adam
SGD learning rate	*choice*[0.01,0.005,0.001, 0.0005,0.0001,0.002,0.003]	-
Adam learning rate	*choice*[0.005,0.001, 0.0005,0.0001,0.00005]	0.0001

Table S5.14: Hyperparameter search space for Tiramisu [7].

S5.14 2D SE U-Net 2D

Template to reproduce the results: SE_U-Net_2D_template.py

Hyperparameter	Search space	Best assignment
Validation	True	True
Random validation	True	True
% of train as validation	10%	10%
Patches	created from train data before network training	created from train data before network training
Patch size	256 × 256	256 × 256
Data augmentation	flips, rotation_range([-180,180])	flips, rotation_range([-180,180])
Number of epochs	360	360
Patience	200	200
Batch size	6	6
Loss type	BCE	BCE
Optimizer	*choice*[SGD,Adam]	SGD
SGD learning rate	0.002	0.002
Adam learning rate	0.0001	-
SE blocks position	*choice*[after each conv, after each conv (but not in bottleneck)]	after each conv (but not in bottleneck)

Table S5.15: Hyperparameter search space for 2D U-Net (adding SE blocks [14]).

S5.15 3D SE U-Net

Template to reproduce the results: SE_U-Net_3D_template.py

Hyperparameter	Search space	Best assignment
Validation	True	True
Random validation	True	True
% of train as validation	10%	10%
Subvolumes	created from train data before network training	created from train data before network training
Subvolume size	$80 \times 80 \times 80$	$80 \times 80 \times 80$
Data augmentation	flips, square_rotations([0,90,180,270]), elastic	flips, square_rotations([0,90,180,270]), elastic
Number of epochs	360	360
Patience	200	200
Batch size	1	1
Loss type	BCE	BCE
Optimizer	Adam	Adam
Adam learning rate	0.0001	0.0001
SE blocks position	*choice*[after each conv, after each conv (but not in bottleneck)]	after each conv (but not in bottleneck)

Table S5.16: Hyperparameter search space for 3D U-Net (adding SE blocks [14]).

S5.16 MultiResUNet [6]

Template to reproduce the results: MultiResUNet_template.py

**Hyperparameter**	**Search space**	**Best assignment**
Validation	True	True
Random validation	True	True
% of train as validation	10%	10%
Patches	created from train data before network training	created from train data before network training
Patch size	256 × 256	256 × 256
Data augmentation	flips, rotation_range([-180,180])	flips, rotation_range([-180,180])
Number of epochs	360	360
Patience	200	200
Batch size	*choice*[1,2,4,6,8,16]	6
Loss type	BCE	BCE
Optimizer	*choice*[SGD,Adam]	Adam
SGD learning rate	*choice*[0.01,0.005,0.001, 0.0005,0.0001,0.002,0.003]	-
Adam learning rate	*choice*[0.005,0.001, 0.0005,0.0001,0.00005]	0.005

Table S5.17: Hyperparameter search space for MultiResUNet [6].

S5.17 MNet [8]

Template to reproduce the results: MNet_template.py

Hyperparameter	Search space	Best assignment
Validation	True	True
Random validation	True	True
% of train as validation	10%	10%
Patches	created from train data before network training	created from train data before network training
Patch size	256×256	256×256
Data augmentation	flips, rotation_range([-180,180])	flips, rotation_range([-180,180])
Number of epochs	360	360
Patience	200	200
Batch size	*choice*[1,2,4,6,8,16]	6
Loss type	BCE	BCE
Optimizer	*choice*[SGD,Adam]	SGD
SGD learning rate	*choice*[0.01,0.005,0.001, 0.0005,0.0001,0.002,0.003]	0.01
Adam learning rate	*choice*[0.005,0.001, 0.0005,0.0001,0.00005]	-

Table S5.18: Hyperparameter search space for MNet [8].

S5.18 3D Vanilla U-Net [11]

Template to reproduce the results: Vanilla_U-Net_3D_template.py

Hyperparameter	Search space	Best assignment
Validation	True	True
Random validation	True	True
% of train as validation	10%	10%
Subvolumes	created from train data before network training	created from train data before network training
Subvolume size	$80 \times 80 \times 80$	$80 \times 80 \times 80$
Data augmentation	flips, square_rotations([0,90,180,270]), elastic	flips, square_rotations([0,90,180,270]), elastic
Number of epochs	360	360
Patience	200	200
Batch size	1	1
Loss type	BCE	BCE
Optimizer	Adam	SGD
Adam learning rate	0.0001	0.0001

Table S5.19: Hyperparameter search space for 3D Vanilla U-Net [11].

S5.19 nnU-Net [9]

Template to reproduce the results: nnU-Net_template.py

Hyperparameter	Search space	Best assignment
Validation	True	True
Random validation	True	True
% of train as validation	10%	10%
Patches	created from train data before network training	created from train data before network training
Patch size	256×256	256×256
Network's final layer activation	*choice*[with sigmoid, with any, with softmax]	without softmax
Data augmentation	flips, rotation_range([-180,180])	flips, rotation_range([-180,180])
Number of epochs	360	360
Patience	200	200
Batch size	*choice*[1,2,4,6,8,16]	4
Loss type	*choice*[BCE,BCE+Dice]	BCE+Dice
Optimizer	*choice*[SGD,Adam]	Adam
SGD learning rate	*choice*[0.05,0.001,0.002]	-
Adam learning rate	*choice*[0.005,0.0005, 0.0001,0.00005]	0.0001

Table S5.20: Hyperparameter search space for nnU-Net [9].

S5.20 U-Net++ [10]

Template to reproduce the results: U-Net++_template.py

Hyperparameter	Search space	Best assignment
Validation	True	True
Random validation	True	True
% of train as validation	10%	10%
Patches	created from train data before network training	created from train data before network training
Patch size	256×256	256×256
Backbone	ResNet50	ResNet50
Data augmentation	flips, rotation_range([-180,180])	flips, rotation_range([-180,180])
Number of epochs	360	360
Patience	200	200
Batch size	*choice*[1,2,4,6,8,16]	1
Loss type	BCE	BCE
Optimizer	*choice*[SGD,Adam]	SGD
SGD learning rate	*choice*[0.0005,0.001, 0.002,0.005,0.01]	0.01
Adam learning rate	*choice*[0.0001, 0.0005,0.001]	-

Table S5.21: Hyperparameter search space for U-Net++ [10]

References

1. H.-C. Cheng and A. Varshney, "Volume segmentation using convolutional neural networks with limited training data," in *2017 IEEE International Conference on Image Processing (ICIP)*. IEEE, 2017, pp. 590–594. 4, 13, 14
2. V. Casser, K. Kang, H. Pfister, and D. Haehn, "Fast mitochondria detection for connectomics," in *Medical Imaging with Deep Learning*, 2020. 4, 15
3. I. Oztel, G. Yolcu, I. Ersoy, T. White, and F. Bunyak, "Mitochondria segmentation in electron microscopy volumes using deep convolutional neural network," in *2017 IEEE International Conference on Bioinformatics and Biomedicine (BIBM)*. IEEE, 2017, pp. 1195–1200. 4, 17
4. C. Xiao, X. Chen, W. Li, L. Li, L. Wang, Q. Xie, and H. Han, "Automatic mitochondria segmentation for EM data using a 3D supervised convolutional network," *Frontiers in Neuroanatomy*, vol. 12, p. 92, 2018. 4, 16
5. J. Dai, Y. Li, K. He, and J. Sun, "R-FCN: Object Detection via Region-based Fully Convolutional Networks," in *Advances in Neural Information Processing Systems*, 2016, pp. 379–387. 5
6. N. Ibtehaz and M. S. Rahman, "MultiResUNet: Rethinking the U-Net architecture for multimodal biomedical image segmentation," *Neural Networks*, vol. 121, pp. 74–87, 2020. 5, 22
7. S. Jégou, M. Drozdzal, D. Vazquez, A. Romero, and Y. Bengio, "The one hundred layers tiramisu: Fully convolutional densenets for semantic segmentation," in *Proceedings of the IEEE Conference on Computer Vision and Pattern Recognition workshops*, 2017, pp. 11–19. 5, 19
8. H. Fu, J. Cheng, Y. Xu, D. W. K. Wong, J. Liu, and X. Cao, "Joint Optic Disc and Cup Segmentation Based on Multi-label Deep Network and Polar Transformation," *IEEE Transactions on Medical Imaging*, vol. 37, no. 7, pp. 1597–1605, 2018. 5, 23
9. F. Isensee, P. F. Jaeger, S. A. Kohl, J. Petersen, and K. H. Maier-Hein, "nnU-Net: a self-configuring method for deep learning-based biomedical image segmentation," *Nature Methods*, vol. 18, no. 2, pp. 203–211, 2021. 5, 25
10. Z. Zhou, M. M. R. Siddiquee, N. Tajbakhsh, and J. Liang, "Unet++: A nested u-Net architecture for medical image segmentation," in *Deep Learning in Medical Image Analysis and Multimodal Learning for Clinical Decision Support*. Springer, 2018, pp. 3–11. 5, 26
11. Ö. Çiçek, A. Abdulkadir, S. S. Lienkamp, T. Brox, and O. Ronneberger, "3D U-Net: Learning Dense Volumetric Segmentation from Sparse Annotation," in *International Conference on Medical Image Computing and Computer-Assisted Intervention*. Springer, 2016, pp. 424–432. 5, 24
12. D. Legland, I. Arganda-Carreras, and P. Andrey, "Morpholibj: integrated library and plugins for mathematical morphology with imagej," *Bioinformatics*, vol. 32, no. 22, pp. 3532–3534, 2016. 6, 7
13. J. Long, E. Shelhamer, and T. Darrell, "Fully convolutional networks for semantic segmentation," in *Proceedings of the IEEE Conference on Computer Vision and Pattern Recognition*, 2015, pp. 3431–3440. 18
14. J. Hu, L. Shen, and G. Sun, "Squeeze-and-excitation networks," in *Proceedings of the IEEE Conference on Computer Vision and Pattern Recognition*, 2018, pp. 7132–7141. 20, 21
